# Sex differences in c‐Fos and EGR‐1/Zif268 activity maps of rat sacral spinal cord following cystometry‐induced micturition

**DOI:** 10.1002/cne.24949

**Published:** 2020-06-11

**Authors:** Nicole M. Wiedmann, Agnes W. Wong, Janet R. Keast, Peregrine B. Osborne

**Affiliations:** ^1^ Department of Anatomy and Neuroscience University of Melbourne Melbourne Victoria Australia

**Keywords:** immediate‐early gene activity mapping, micturition, parasympathetic preganglionic neuron, RRID:AB_10609634, RRID:AB_11214092, RRID:AB_2097174, RRID:AB_2313584, RRID:AB_2340813, RRID:AB_2533990, RRID:AB_390204, RRID:SCR_000432, RRID:SCR_001905, sacral preganglionic nucleus, sacral spinal cord, urinary bladder

## Abstract

Storage and voiding of urine from the lower urinary tract (LUT) must be timed precisely to occur in appropriate behavioral contexts. A major part of the CNS circuit that coordinates this activity is found in the lumbosacral spinal cord. Immediate early gene (IEG) activity mapping has been widely used to investigate the lumbosacral LUT‐related circuit, but most reports focus on the effects of noxious stimulation in anesthetized female rats. Here we use c‐Fos and EGR‐1 (Zif268) activity mapping of lumbosacral spinal cord to investigate cystometry‐induced micturition in awake female and male rats. In females, after cystometry c‐Fos neurons in spinal cord segments L5–S2 were concentrated in the sacral parasympathetic nucleus (SPN), dorsal horn laminae II–IV, and dorsal commissural nucleus (SDCom). Comparisons of cystometry and control groups in male and female revealed sex differences. Activity mapping suggested dorsal horn laminae II–IV was activated in females but showed net inhibition in males. However, inhibition in male rats was not detected by EGR‐1 activity mapping, which showed low coexpression with c‐Fos. A class of catecholamine neurons in SPN and SDCom neurons were also more strongly activated by micturition in females. In both sexes, most c‐Fos neurons were identified as excitatory by their absence of Pax2 expression. In conclusion, IEG mapping in awake male and female rats has extended our understanding of the functional molecular anatomy of the LUT‐related circuit in spinal cord. Using this approach, we have identified sex differences that were not detected by previous studies in anesthetized rats.

## Graphical abstract

A major part of the circuit that coordinates bladder voiding is found in the lumbosacral spinal cord. Here we use c‐Fos and EGR‐1 (Zif268) activity mapping of lumbosacral spinal cord to investigate cystometry‐induced micturition in awake female and male rats. In both sexes, most c‐Fos neurons were identified as excitatory by their absence of Pax2 expression. Using this approach, we have identified sex differences that were not detected by previous studies in anesthetized rats.

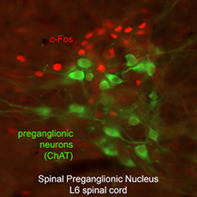

**Abbreviations**
A‐LTMRs: A low‐threshold mechanoreceptors
ChAT: choline acetyltransferase
C‐LTMRs: C low‐threshold mechanoreceptors
EGR‐1: early growth response protein 1
IEG: immediate early gene
LUT: lower urinary tract
Pax2: paired box gene 2
SDCom: dorsal commissural nucleus
SPN: sacral preganglionic nucleus
TH: tyrosine hydroxylase

## 1 INTRODUCTION

Micturition (voiding of urine) and scent marking are normally precisely timed to synchronize with appropriate behaviors. This control is provided by a neural circuit that is external to the lower urinary tract (LUT) and incorporates (a) peripheral sensory, autonomic motor, and somatomotor projections to the bladder, urethra and external urethral sphincter (EUS); (b) LUT‐related networks in spinal cord; and (c) bidirectional connections with an extensive distributed brain network that integrates autonomic function with behavioral controls (Fowler, Griffiths, & de Groat, 2008; Holstege & Collewijn, 2009).This extended neural network provides the flexibility needed to integrate LUT control with complex physiological and behavioral demands. For example, it can adaptively regulate storage of urine in the context of behaviors that maintain fluid balance in response to interoceptive cues (Beckel & Holstege, 2011; de Groat & Wickens, 2013; Owens, Allen, Ondobaka, & Friston, 2018). It also can initiate voiding and scent marking in the context of social, defensive and sexual behaviors triggered by exteroceptive cues. These complex functions have stimulated renewed interest (Hou, Hyun, et al., 2016; Keller et al., 2018; Yao et al., 2018) in the functional organization of this extended visceral sensorimotor circuit processes sensory information encoding the state of the LUT to produce synchronized LUT activity in the appropriate behavioral context.

The LUT receives sensory and motor projections from both the lumbosacral and thoracolumbar regions of spinal cord situated either side of the lumbar enlargement (de Groat, Griffiths, & Yoshimura, 2015; Holstege & Collewijn, 2009). However, strong evidence suggests the lumbosacral spinal cord is the primary controller of voiding. It coordinates activation of the sacral parasympathetic motor pathway to the urinary bladder with reciprocal inhibition of the somatomotor pathway to the urethral rhabdosphincter (de Groat & Wickens, 2013). In rodents, L6 and S1 spinal cord segments are the primary source of sensory and motor projections to the LUT (Applebaum, Vance, & Coggeshall, 1980; Nadelhaft & Vera, 1996; Nance, Burns, Klein, & Burden, 1988; Vera & Nadelhaft, 1992, 2000). Mechanosensory information encoding bladder fullness is provided by A‐delta low‐threshold mechanosensor (A‐LTMRs) fibers, but in rodents, C‐LTMRs may also contribute (Janig & Koltzenburg, 1990; Sengupta & Gebhart, 1994; Shea, Cai, Crepps, Mason, & Perl, 2000). LUT functions such as micturition or scent marking are controlled by descending motor commands from Barrington's nucleus (pontine micturition center) supported by other brain regions (Hou, Hyun, et al., 2016; Keller et al., 2018; Yao et al., 2018). This input is received by multiple types of interneurons (local circuit neurons) that only project within or between spinal cord segments (Deuchars, 2011; Shefchyk, 2001; Vera & Nadelhaft, 2000), in addition to direct input onto parasympathetic preganglionic neurons that provide the autonomic motor output required to contract the bladder and relax the internal smooth muscle sphincter during voiding (Anderson, Keast, & McLachlan, 2009; Holstege & Collewijn, 2009). In rat, these neurons are located in the sacral parasympathetic nucleus (SPN) and project to autonomic (parasympathetic) postganglionic neurons in the bilateral pelvic ganglia. Activity in the autonomic motor pathway must be coordinated with relaxation of the striated muscle rhabdosphincter, which is controlled by sacral somatomotor neurons (Holstege & Collewijn, 2009; Thor & de Groat, 2010). Activation of the LUT spinal cord circuit must also be coordinated with appropriate activities of other pelvic viscera (such as the colon and sex organs) and somatic structures such as the pelvic floor, hindlimbs, and in rat, the tail (Merkulyeva et al., 2019; Thor & de Groat, 2010).

Immediate early gene (IEG) activity mapping has been widely used to identify spinal cord neurons in LUT‐related circuits. However, these reports have mostly focused on acute noxious LUT stimulation or models of visceral hyperalgesia. Furthermore, in almost all cases the experiments have been performed under anesthesia, which alters the activity of LUT‐related neural circuits and removes circuits active in behaving awake rats. IEG‐mapping studies of non‐noxious stimulation also only report on female subjects, even though the sacral LUT‐related circuit controls sexually dimorphic organs and functions in sex‐specific behavior. Here, we used IEG activity mapping to study non‐noxious LUT stimulation by cystometry‐induced micturition in awake female and male rats.

## 2 METHODS

### 2.1 Animals

All procedures were approved by the Animal Ethics Committee of the University of Melbourne and complied with the Australian Code for the Care and Use of Animals for Scientific Purposes (National Health and Medical Research Council of Australia). Twelve male and twelve female adult Sprague‐Dawley rats (Biomedical Sciences Animal Facility, University of Melbourne) aged 8–9 weeks were used in this study. In female rats, estrous cycle was not measured and could increase between‐subject variance when compared to males. Rats were housed individually with environmental enrichment under a 12‐hr light–dark cycle with ad libitum access to food and water.

### 2.2 Apparatus

All rats were housed individually under identical conditions over the 10 days period preceding the final IEG activity mapping session. On the final 3 days they were habituated for 30 min to the apparatus in a dedicated room. Each rat was placed unrestrained in a clear Perspex recording box (20.5 × 20.5 × 14 cm), which had a perforated steel mesh floor and was elevated on a 45 cm high frame. Rats implanted with a bladder cannula were also connected to the infusion system used for cystometry during habituation. Control rats were handled for an equivalent time before being placed in the recording box. Habituation and the final activity mapping session were confined to the morning to minimize effects of diurnal variation.

### 2.3 Surgery

For cystometry, bladder catheters were implanted under isoflurane anesthesia (3% for induction and 1.8–2% for maintenance). A midline incision in the lower abdomen was made to expose the apex of the bladder dome. This was punctured with an 18G needle so that a polyethylene catheter (PE‐10: od: 0.61 mm × id: 0.28 mm; SteriHealth, VIC, Australia) with a flared end (made by heating the tubing) could be inserted and secured with a purse‐string suture (sterile monofilament suture II PDS [polydioxanone]; Ethicon, Somerville, NJ). The length of the catheter was then passed through a subcutaneous tunnel and externalized by anchoring to the interscapular skin. The free end of the catheter was sealed to prevent leakage. The incision in the abdominal wall was then sutured, and the skin incision closed with surgical skin staples (Fine Science Tools, Foster City, CA). Perioperative analgesia was provided by administering buprenorphine (0.5 mg/kg, s.c.) prior to surgery and ~10 hr postsurgery. Following surgery, the catheter was infused daily with 0.9% saline (0.5 ml) and gentamicin (0.2 ml, 40 mg/ml) for 3 days, and with 0.9% saline alone for 7 days.

### 2.4 Continuous cystometry

**FIGURE 1:**
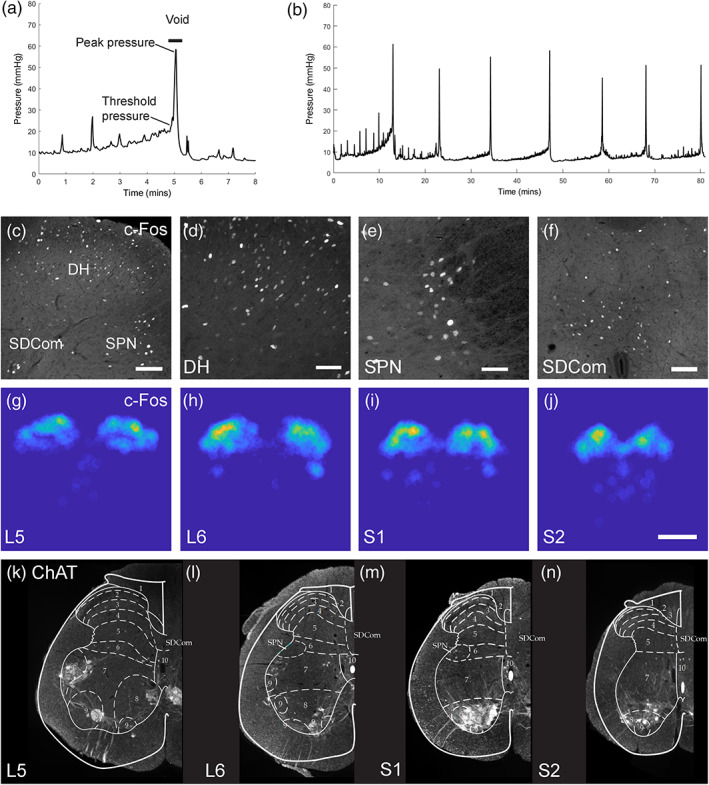
Activity mapping following cystometry in awake rats. Example cystometrograms from awake male rats showing (a) single, or (b) multiple micturition cycles; with examples of nonvoiding bladder contractions that typically increase in frequency and amplitude over the course of the filling phase preceding voiding contractions. Also shown are example measurements used to quantify cystometric parameters. (c) c‐Fos^+^ neurons in a dorsal quadrant of L6 spinal cord are shown at higher magnification in dorsal horn (2Sp, 3Sp, and 4Sp) (d), sacral preganglionic nucleus (e) and sacral dorsal commissural nucleus (f). Heat maps (g–j) of the spatial distribution of c‐Fos^+^ neurons averaged across five sections in spinal cord segments L5–S2 in a male rat. (k–n) Transverse sections of the lumbosacral spinal cord segments L5–S2 showing ChAT^+^ neurons in the sacral preganglionic nucleus (SPN) with transverse maps overlaid (modified from [Molander, Xu, & Grant, 1984]) used to define ROIs for IEG neuron counts in: Lamina I (1Sp), lamina II (2Sp), lamina III (3Sp), lamina IV (4Sp), sacral dorsal commissural nucleus (SDCom), lamina V (5Sp), lamina VI (6Sp), sacral preganglionic nucleus (SPN), lamina VII (7Sp), lamina VIII (8Sp), and lamina X (10Sp). Scale bars: 100 μm (c,f), 50 μm (d,e), 500 μm (g‐j) [Color figure can be viewed at wileyonlinelibrary.com]

To induce repeated micturition, the bladder catheter of rats in the *Cystometry* groups was connected to an in‐line pressure transducer (MLT0670, ADInstruments, NSW, Australia) and syringe pump (Harvard Apparatus, Holliston, MA). This was used for continuous cytometry by infusing sterile room temperature saline into the bladder at a rate of 0.1 ml/min. Cystometry was performed during the last habituation period and in the final session, and was digitally recorded (PowerLab 4/26, ADInstruments, NSW, Australia). Urodynamic parameters (Andersson, Soler, & Füllhase, 2011) were measured over a 90 min period after allowing 30 min for the voiding pattern to stabilize: intervoiding interval, peak pressure (the maximal bladder pressure during voiding), void duration (time between threshold pressure and return to baseline), and threshold pressure (Figure 1a). Threshold pressure was defined as the pressure required for the bladder to contract and subsequently void. Each of the pressure measurements was expressed relative to the baseline bladder pressure.

### 2.5 IEG activity mapping experiment

The experimental design used six female and six male rats assigned to *Cystometry* and *Control* groups (*n* = 6 per group, *n* = 24 in total). Each rat was placed unrestrained in a test box for 2 hr, during which micturition was induced in the *Cystometry* groups by performing continuous cystometric recording. All rats were then returned to their home cage for 2 hr, before being anesthetized (ketamine 100 mg/kg and xylazine 10 mg/kg, i.p.) and fixed (intracardiac infusion of approximately 100 ml of 1% sodium nitrite and 5,000 IU/ml heparin in 0.9% saline, followed by approximately 500 ml of 4% paraformaldehyde in 0.1 M phosphate buffer, pH 7.4). This period of 2 hr after the final cystometry test session was required to maximize activity‐dependent translation of c‐Fos protein (Yap & Greenberg, 2018).

### 2.6 Immunohistochemistry

After perfusion with saline, the spinal cord was removed and the lumbosacral spinal segments (L5 – S2) subdissected and postfixed for 1 hr in the same fixative. After three 1 hr washes in 0.1 M phosphate‐buffered saline (PBS), pH 7, the tissue was incubated overnight in 0.1 M PBS containing 30% sucrose and embedded in an inert mounting medium (OCT; Tissue‐Tek, Sakura, Torrance, CA). Frozen sections (40 μm) were then cut in the transverse plane and collected as four 1:4 series (160 μm between sections) spanning the spinal cord segments L5–S2. Five sections per spinal segment were labeled with each antibody combination of interest. Free‐floating sections were washed in 0.1 M PBS (pH 7.2) before being incubated for 2 hr in 0.1 M PBS containing 10% nonimmune horse serum (NHS; Sigma‐Aldrich) and 0.5% Triton X‐100. To characterize expression patterns, sections were incubated for 48–72 hr at room temperature with the following combinations of primary antisera (Table 1): c‐Fos (1:100) with either EGR‐1 (1:5,000); Pax2 (1:1,000); TH (1:2,000); or ChAT (1:500). Each antibody combination was prepared in PBS containing 0.1% sodium azide, 2% NHS, and 0.5% Triton X‐100. After washes in PBS, sections were then incubated for 4 hr at room temperature with Cy3‐labeled donkey anti‐mouse (Jackson Immunoresearch, West Grove, PA; 715‐165‐150; batch 89001; RRID:AB_2340813; 1:2,000) and AF488‐labeled donkey anti‐rabbit (Jackson Immunoresearch; 711‐545‐152; batch 134352; RRID:AB_2313584; 1:1,000). Sections were then washed in PBS, mounted onto glass slides, and cover‐slipped with carbonate‐buffered glycerol (pH 8.6).

**TABLE 1:** Primary antibodies used for immunohistochemistry

Antigen	Description of immunogen	Supplier/catalogue number/RRID	Dilution
c‐Fos	Affinity purified mouse monoclonal antibody specific for an epitope mapping between amino acids 120–155 within an internal region of c‐Fos of human origin	Santa Cruz Biotechnology, Inc., Santa Cruz, CA; (E‐8) sc‐166940; batch D2318; RRID:AB_10609634	1:100
EGR‐1/Zif268	Affinity purified rabbit polyclonal for epitope mapping at the C‐terminus of the EGR‐1/Zif268 of human origin	Santa Cruz Biotechnology, Inc., Santa Cruz; (588) sc‐110; batch K1714; RRID:AB_2097174	1:5,000
Tyrosine hydroxylase (TH)	Affinity purified rabbit polyclonal antibody generated in rabbit against the human TH. Western blot analysis detects a band of approximately 62 kD	Millipore, Temecula, CA; AB152; batch 2639432; RRID:AB_390204	1:2,000
ChAT	Immunoaffinity purified polyclonal antibody generated in goat against the human placental enzyme	Millipore, Temecula, CA; AB144P; batch 2929343; RRID:AB_11214092	1:500
Pax2	Affinity purified rabbit polyclonal specific for the GST‐Pax‐2 fusion protein derived from the C‐terminal domain (aa188‐385) of the murine Pax‐2 protein	Invitrogen, Carlsbad, CA; 71‐6000; batch RE233286; RRID:AB_2533990	1:1,000

### 2.7 Antibody characterization

Primary antibodies were obtained from the following sources, noting that information of specificity has been provided by the manufacturers:

1. c‐Fos (Santa Cruz Biotechnology, Inc., Santa Cruz, CA; (E‐8) sc‐166940; batch D2318; RRID:AB_10609634): affinity purified mouse monoclonal antibody specific for an epitope mapping between amino acids 120–155 within an internal region of c‐Fos of human origin. Western blot analysis detects a band of approximately 62 kDa.
2. EGR‐1 (Santa Cruz Biotechnology, Inc.; (588) sc‐110; batch K1714; RRID:AB_2097174): affinity purified rabbit polyclonal for epitope mapping at the C‐terminus of the EGR‐1 of human origin.
3. TH (Millipore, Temecula, CA; AB152; batch 2639432; RRID:AB_390204): affinity purified rabbit polyclonal antibody generated in rabbit against the human TH. Western blot analysis detects a band of approximately 62 kD.
4. ChAT (Millipore, Temecula; AB144P; batch 2929343; RRID:AB_11214092): immunoaffinity purified polyclonal antibody generated in goat against the human placental enzyme. Western blot analysis of NIH/3T3 lysates detects a band at 70 kDa, consistent with the predicted weight of ChAT protein.
5. Pax2 (Invitrogen, Carlsbad, CA; 71‐6000; batch RE233286; RRID:AB_2533990): affinity purified rabbit polyclonal specific for the GST‐Pax‐2 fusion protein derived from the C‐terminal domain (aa188‐385) of the murine Pax‐2 protein. Western blot analysis detects a band of approximately 45 kDa.

Secondary antisera were raised in donkey and obtained from the following suppliers:

1. Anti‐mouse Cy3 (Jackson Immunoresearch, West Grove, PA; 715‐165‐150; batch 89001; RRID:AB_2340813).
2. Anti‐rabbit AF488 (Jackson Immunoresearch; 711‐545‐152; batch 134352; RRID:AB_2313584).

### 2.8 Imaging and quantification

For each antibody combination, spinal cord sections (L5–S2; five sections per segmental level) were anatomically ordered from 1 to 20. Entire transverse sections were imaged (tile scanned at 12 Bit, pixel scaling 0.645 μm × 0.645 μm) using a Zeiss AxioImager M2 (Zeiss, Oberkochan, Germany). In each section, positive neurons were counted across spinal cord regions, defined by the boundaries described in (Watson, Paxinos, & Kayalioglu, 2009), and the sacral preganglionic nucleus (SPN) outlined in (Forrest, Payne, Keast, & Osborne, 2015). This was done by superimposing and warping (in *X* and *Y* axis only) the schematics outlining each lamina over the relative section. Each template was positioned using lamina III and the central canal as fiduciary markers. As the schematics did not include the SPN, this boundary was defined by and superimposing schematics onto ChAT immunolabeled transverse sections (a marker of preganglionic neurons in the SPN, Figure 1c,d), and using the distribution of ChAT neurons to define the SPN boundary as shown in Figure 1. Neurons were counted using ImageJ FIJI Cell Counter plugin, where a marker (denoting an *xy* coordinate) was designated for each positive cell. To avoid double‐counting neurons, every fourth section was analyzed for each antibody combination (160 μm between sections).

### 2.9 Statistical analysis

All visual and statistical summaries and comparisons of data were performed using R Project for Statistical Computing (Version 3.5.2; RRID:SCR_001905) and RStudio (Version 1.1.4; RRID:SCR_000432). Data were analyzed using an estimation statistics framework (Calin‐Jageman & Cumming, 2019). The R package function ggplot2::stat_smooth was used for general additive modeling (GAM) with a negative‐binomial distribution (Wood, 2017). This was used to simultaneously fit conditional means and 95% CIs to neuron counts and densities measured in ordered series of sections through L6‐S1 spinal cord, with Sex (female and male) and *Group* (cystometry and control) as factors. Two‐sample comparisons were made using exact Welsh two sample *t*‐tests to estimate means, 95% CIs and *P* values. Corrections for multiple testing were made using the Hommel step‐up modification of the Bonferroni procedure (Blakesley et al., 2009).

### 2.10 Figure preparation

Monochrome images were digitally colorized and where necessary adjustments made in contrast and brightness to best represent the immunostaining as seen under the microscope (Adobe InDesign and Photoshop CC; Adobe Systems, San Jose, CA). Heat maps of neuronal distribution from XY coordinates were generated using MATLAB version R2017b.

## 3 RESULTS

### 3.1 c‐Fos activity mapping after cystometry in awake rats

All rats were habituated to the experimental apparatus for 3 days pretest to minimize behavioral activation due to handling and exposure to a novel test environment. For activity mapping, continuous cystometry (flow rate: 0.1 ml/min) was used as a non‐noxious mechanical stimulus to induce repeated micturition cycles over 2 hr (Figure 1a,b). Standard cystometric parameters (Figure 1a) measured in the *Cystometry* group during this period (Table 2) were all within the normal range reported by previous studies using awake rats (Andersson et al., 2011). This suggested there were minimal or no carry‐over effects of surgery causing hyperactivity after 10 days (e.g., short intervoid interval).

**TABLE 2:** Urodynamic parameters^a^ measured by continuous cystometry in awake rats

	Male (*n* = 6)^b^	Female (*n* = 6)	*p* ^c^
Threshold pressure (mmHg)^d^	4.3 ± 0.9^e^	7.6 ± 2.1	.188
Peak pressure (mmHg)^d^	44.8 ± 11.4	51.8 ± 20.6	.773
Intervoid interval (min)	8.4 ± 1.0	6.7 ± 0.9	.237
Void duration (s)	36.1 ± 4.1	30.2 ± 1.2	.212

^a^ Andersson et al. (2011).

^b^ *n*, number of rats.

^c^ Significance determined by independent samples *t*‐test.

^d^ Relative to baseline (minimum) pressure.

^e^ Mean ± SEM.

Activity‐dependent translation of c‐Fos protein in sections of lumbosacral cord was detected by immunohistochemical staining of c‐Fos^+^ neuronal nuclei (Figure 1c–f). To visualize how these c‐Fos neurons were distributed, we first used spinal cords from a female and male rat in the cystometry group to produce heat maps by averaging locations across five sections per segment (L5–S2) in one spinal cord (Figure 1g–j shows data for male rat; female data not shown). This showed the highest concentration of c‐Fos neurons was in dorsal horn laminae II and III from which a graded distribution spread ventrally into the lateral and medial midline region of spinal cord segments L6 and S1.

For quantitative comparisons by experimental group and sex, we used the activated areas shown by the heat maps in segments L5–S2 to guide selection of 11 regions of interest (ROIs). These were identified using a rat spinal cord atlas (Watson et al., 2009) to provide four templates aligning to each spinal cord segment. Each template was positioned using lamina III and the central canal as fiduciary markers. Templates were validated by fluorescent Nissl and immunostaining for choline acetyltransferase (ChAT) (Figure 1k–n), which confirmed that ChAT^+^ sacral preganglionic neurons clustered in the SPN region defined by the template. By using this approach, we were able to increase the rostrocaudal coverage and spatial resolution of our analysis relative to most previous IEG mapping studies. These most commonly analyze four large ROIs—corresponding to medial, and lateral regions of the upper dorsal horn; and two midline areas centered on the SPN and the dorsal commissural nucleus (SDCom) (Birder & de Groat, 1992a, 1993; Kakizaki, Yoshiyama, & de Groat, 1996; Vizzard, 2000a).

### 3.2 Sex differences in sacral spinal cord activation by cystometry‐induced micturition

**FIGURE 2:**
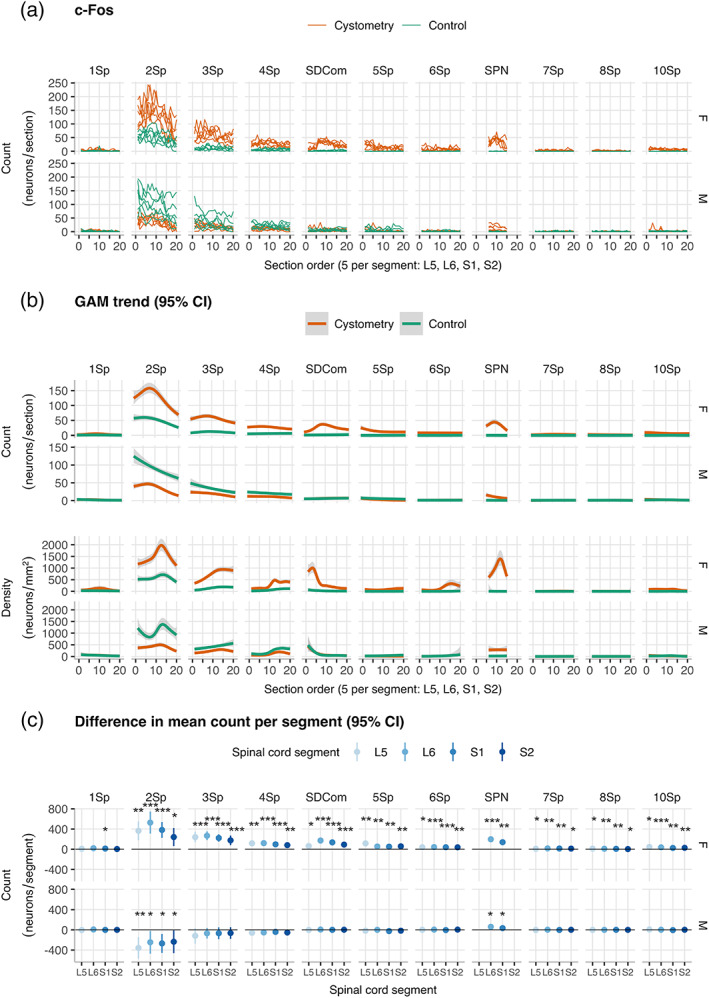
Spatial analysis of c‐Fos^+^ neuron counts in lumbosacral spinal cord in male and female rats. (a) Region of interest (ROI) neuron counts by subject in *Cystometry* and *Control* groups of male and female rats (*n* = 6 per group, *n* = 24 total). Plots show rostrocaudal distribution over ordered sections across spinal cord segments L5–S2 (five sections per segment, 40 μm thick, 160 μm interval). ROIs: Lamina I (1Sp), lamina II (2Sp), lamina III (3Sp), lamina IV (4Sp), sacral dorsal commissural nucleus (SDCom), lamina V (5Sp), lamina VI (6Sp), sacral preganglionic nucleus (SPN), lamina VII (7Sp), lamina VIII (8Sp), and lamina X (10Sp). (b) Comparison of conditional smoothed means and 95% CIs estimated by negative binomial generalized additive modeling (GAM) of c‐Fos^+^ activity mapping in *Cystometry* and *Control* female and male rats. Plots in the upper panel show smoothed trends simultaneously fit to region of interest (ROI) counts versus ordered sections over spinal cord segments L5–S2. Plots in the lower panel shown trends from fits to approximate neuron densities estimated by normalizing to the areas of the ROIs in the counting templates. (c) Plots of difference in means and 95% CI for neurons counts in *Cystometry* and *Control* groups by spinal cord segment (*n =* 6 male and female rats per group). *p < .05, **p < .01, ***p < .001; Walsh Exact Test with Hommel correction for multiplicity [Color figure can be viewed at wileyonlinelibrary.com]

We next performed a spatial analysis comparing regional and segmental c‐Fos^+^ neuron counts (Figure 2) in *Cystometry* and *Control* groups of female and male rats. To determine baseline IEG activity and maximize the response window for activity mapping, the subjects in the *Control* group were surgically naive rats that were age matched and received the same handling and exposure to the experimental apparatus.

Plotting the rostrocaudal distribution of c‐Fos^+^ neuron counts by ROI (Figure 2) provided a visual comparison of the relative rostrocaudal and regional activity in female and male rats in group *Cystometry*. The overall pattern of c‐Fos^+^ neuron activity was relatively uniform across subjects within each sex (Figure 2a). In both sexes, the highest neuron counts were in SPN, dorsal horn and SDCom. In the dorsal horn c‐Fos counts showed graded reductions extending from lamina II ventrally to lamina IV and rostrocaudally to spinal cord segment S2.

To visualize possible group and sex differences, we used negative binomial generalized additive modeling (GAM) to simultaneously fit smoothed trends, and estimate conditional means and 95% CIs for each experimental group (Figure 2b). As the ROIs were also different in size, we also fit smooths to neuron densities that were estimated by normalizing neuron counts, using a weighting based on the estimated area of each ROI (Figure 2b, lower panel).

In females, the dorsal horn laminae, SDCom, and SPN were maximally activated (highest neuron count) in segment L6, which is the spinal cord segment that receives most of the central axons projecting from LUT primary afferents (de Groat & Yoshimura, 2010). In the dorsal horn, c‐Fos was most strongly activated in lamina II but showed a graded decrease across the adjacent laminae III and IV. The control group showed some, but reduced, baseline activation of the laminae II and III compared to the *Cystometry* group, whereas all other regions showed minimal or no baseline activity. The normalized data (Figure 2b, lower panel) revealed a slightly different pattern, with maximal activation shifting caudally to S1 in dorsal horn and SPN, but rostrally to L5 in SDCom.

In males, the SPN was activated by cystometry but this response was reduced in comparison to females. In further contrast to females, no activation was detected in SDCom of male rats following cystometry. In male rats, there was also no activation detected in the dorsal horn after cystometry and instead a net decrease in c‐Fos induction relative to controls was observed. Potentially related to this observation, c‐Fos counts in segments L5 and S2 in the unstimulated male control group and females after cystometry were similar. This higher level of baseline c‐Fos in the dorsal horn of males may have impacted on our capacity to detect an effect of cystometry.

Two group comparisons were made using neuron counts per segment as a summary variable and plotting the mean and CI 95% of the mean differences for visual comparison (Figure 2c). In females, lamina I (1Sp) was the only region where cystometry did not increase c‐Fos^+^ neuron counts in two or more spinal cord segments. However, proportionally, the largest differences in means (effect size) were detected in laminae II–IV of dorsal horn (2Sp, 3Sp, and 4Sp); the sacral dorsal commissural nucleus (SDCom); and the SPN. By contrast, in males, activation was only detected in SPN; cystometry was found to have no effect in SDCom and caused a statistically significant *reduction* in activity in dorsal horn lamina II (2Sp).

### 3.3 EGR‐1 induction reveals activation of dorsal horn following cystometry in male rats

**FIGURE 3:**
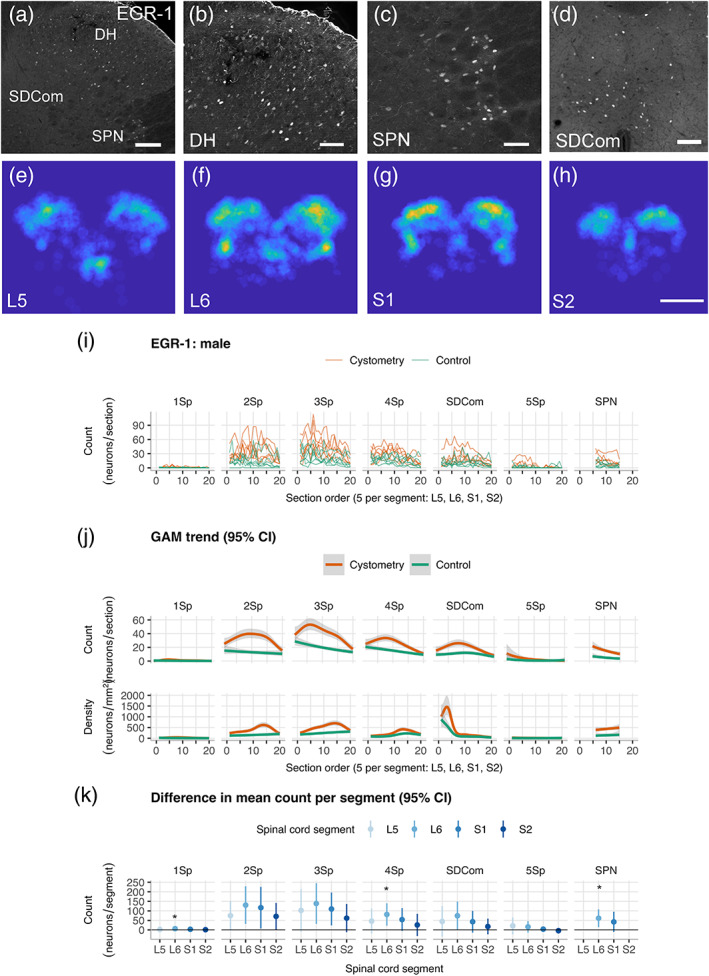
Spatial analysis of EGR‐1^+^ neuron counts in lumbosacral spinal cord in male rats. (a–d) EGR‐1^+^ neurons in a dorsal quadrant of L6 spinal cord (a) are shown at higher magnification in dorsal horn (2Sp, 3Sp, and 4Sp) (b), sacral preganglionic nucleus (c) and sacral dorsal commissural nucleus (d). Heat maps (e–h) of the spatial distribution of EGR‐1^+^ neurons averaged across five sections in spinal cord segments L5–S2 in a male rat. (i) Region of interest (ROI) neuron counts by subject in *Cystometry* and *Control* groups of male rats (*n* = 6 per group, *n* = 12 total). Plots show rostrocaudal distribution over ordered sections across spinal cord segments L5 to S2 (5 sections per segment, 40 μm thick, 160 μm interval). ROIs: Lamina I (1Sp), lamina II (2Sp), lamina III (3Sp), lamina IV (4Sp), sacral dorsal commissural nucleus (SDCom), lamina V (5Sp), lamina VI (6Sp), sacral preganglionic nucleus (SPN), lamina VII (7Sp), lamina VIII (8Sp), and lamina X (10Sp). (j) Comparison of conditional smoothed means and 95% CIs estimated by negative binomial generalized additive modeling (GAM) of c‐Fos^+^ activity mapping in *Cystometry* and *Control* male rats. Plots in the upper panel show smoothed trends simultaneously fit to region of interest (ROI) counts versus ordered sections over spinal cord segments L5–S2. Plots in the lower panel shown trends from fits to approximate neuron densities estimated by normalizing to the areas of the ROIs in the counting templates. (k) Plots of difference in group means and 95% CI for neurons counts in *Cystometry* and *Control* groups by spinal cord segment (*n =* 6 male rats per group). Scale bars: 100 μm (a,d), 50 μm (b,c), 500 μm (e–h). *p < .05, Walsh Exact Test with Hommel correction for multiplicity [Color figure can be viewed at wileyonlinelibrary.com]

The outcome of our c‐Fos activity mapping experiment identified an unexpected sex difference associated with a net reduction or inhibition of dorsal horn activity following induction of micturition in awake male rats. To explore this further, we used another series of sections from the same male rats for activity mapping with EGR‐1 (Zif268) (Figure 3a–d). This is another IEG protein that functions as an activity‐dependent transcription factor in neurons (Yap & Greenberg, 2018). Previous comparisons of EGR‐1 and c‐Fos activity mapping have demonstrated different patterns of spinal cord activation can result from peripheral sensory stimulation (Densmore, Kalous, Keast, & Osborne, 2010; Ko et al., 2005; Lanteri‐Minet et al., 1993; Lanteri‐Minet, Bon, de Pommery, Michiels, & Menetrey, 1995; Rahman et al., 2002; Yap & Greenberg, 2018). Following cystometry in awake male rats, the rostrocaudal and regional distribution of EGR1^+^ neurons shown by heat maps (Figure 3e–h) and spatial analysis (Figure 3i–k) showed no evidence of cystometry‐induced inhibition of dorsal horn, as the smoothed trends (Figure 3i) and effect sizes (Figure 3k) were qualitatively similar to the pattern seen with c‐Fos. Comparison of cystometry and control groups adjusted for multiple testing also showed statistically significant increases in EGR‐1^+^ neurons in SPN and dorsal horn lamina IV.

**FIGURE 4:**
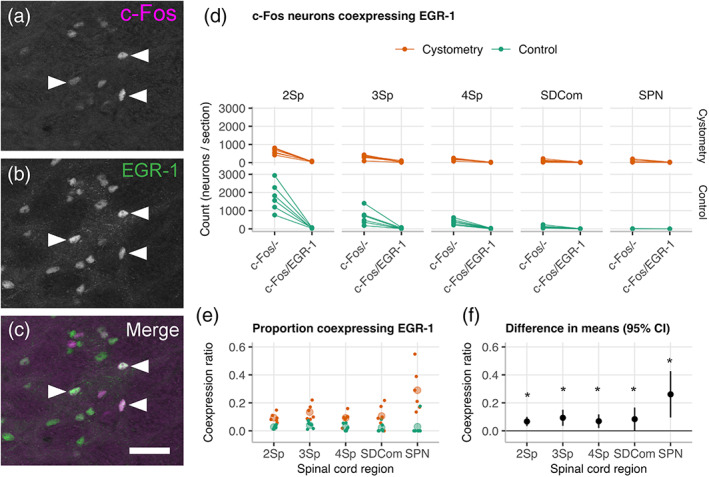
Analysis of EGR‐1 coexpression in c‐Fos^+^ neurons in male rats. (a–c) Images showing coexpression (arrows) of EGR‐1^+^ and c‐Fos^+^ in nuclei of neurons distributed in lamina II (2Sp) of a transverse section of L6 spinal cord in a male rat. EGR‐1 activation in several c‐Fos^+^ lumbosacral spinal cord neurons. (d) EGR‐1^+^ and EGR‐1^+^/c‐Fos^+^ ROI neuron counts and (e) relative proportions across 2Sp, lamina III (3Sp), lamina IV (4Sp) of the dorsal horn; sacral dorsal commissural nucleus (SDCom); and sacral preganglionic nucleus (SPN) in naïve and after cystometry in male rats (*n* = 6 per group) showing low coexpression. Comparisons of the difference in group means and 95% CIs (f) showed small but statistically significant increase in coexpression in the cystometry group. *P < .05, Walsh Exact Test with Hommel correction for multiplicity [Color figure can be viewed at wileyonlinelibrary.com]

We next used co‐labelling of EGR1 and c‐Fos to determine if they were detecting activity in independent or overlapping populations of spinal cord neurons in male rats. Initial inspection of sections from the *Cystometry* group indicating a low level of coexpression (Figure 4a–c). Quantification in the *Control* group (Figure 4d–f) showed the coexpression of EGR‐1^+^ and c‐Fos^+^ to be negligible, noting that in these tissues there were very few EGR‐1^+^ neurons. Comparison with the *Cystometry* group detected statistically‐significant increases in co‐expression in 2Sp, 3Sp, 4Sp, SDCom, and SPN (Figure 4), with the largest effect occurring in SPN (Figure 4e,f).

### 3.4 c‐Fos is co‐expressed by tyrosine hydroxylase+ neurons but not sacral preganglionic neurons in SPN

**FIGURE 5:**
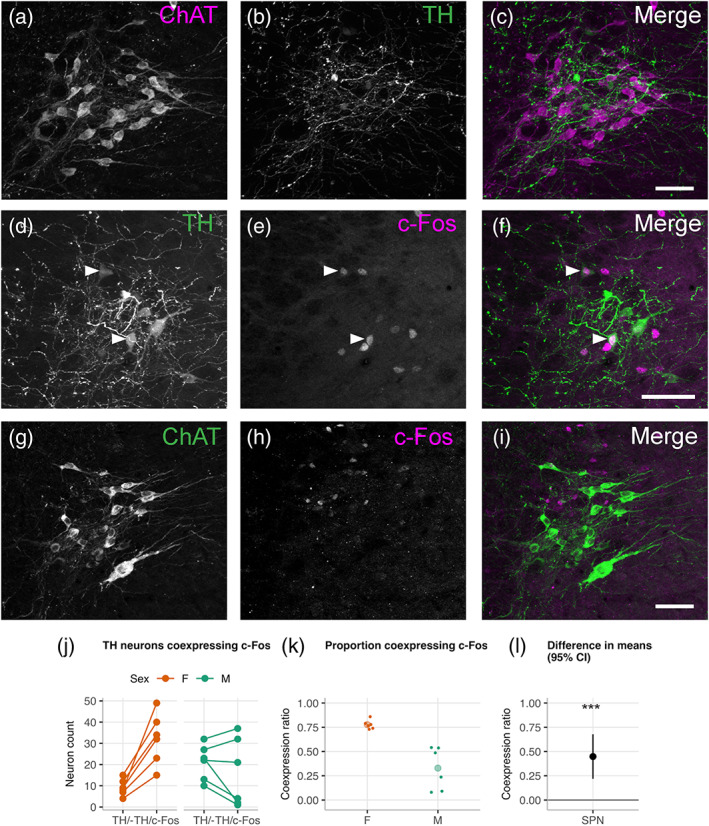
c‐Fos activation in TH^+^ and ChAT^+^ neurons in the sacral preganglionic nucleus (SPN) of awake female and male rats following cystometry. (a–c) Image of SPN showing distinct nonoverlapping populations of TH^+^ neurons and ChAT^+^ sacral parasympathetic preganglionic neurons, surrounded by a dense plexus of TH^+^ terminals. (d–f) After cystometry, c‐Fos was coexpressed in both TH^+^ and TH^−^ neurons, but not in ChAT^+^ sacral parasympathetic preganglionic neurons (g–i). (j) Plots of ROI neuron counts and (k) ratios comparing c‐Fos activation in SPN TH^+^ neurons in female and male rats. (l) The difference in group mean and 95%CIs suggested a higher proportion of TH^+^ neurons are activated in females following cystometry (*p*‐value = .003). Scale bar = 20 μm. ***p < .001; Walsh Exact Test with Hommel correction for multiplicity [Color figure can be viewed at wileyonlinelibrary.com]

In rat, the SPN contains a class of tyrosine hydroxylase (TH^+^) catecholamine neurons, which are suggested to regulate micturition by synthesizing and releasing dopamine as the effector neurotransmitter (Hou, Carson, et al., 2016; Mouchet et al., 1986; Mouchet, Manier, & Feuerstein, 1992). The presence of c‐Fos^+^/TH^+^ neurons in SPN following cystometry in anesthetized female rats (Hou, Carson, et al., 2016) suggests these neurons are activated by micturition. In this study, we determined that c‐Fos^+^/TH^+^ neurons are also present in the SPN following cystometry in awake rats (Figure 5a–f). A comparison by sex showed the percentage of c‐Fos^+^/TH^+^ neurons was significantly higher in female (78%) than male (33%) rats (Welch two sample *t*‐test: *P* = .004, *df* = 10; Figure 5k–m).

The SPN contains all of the LUT parasympathetic preganglionic cholinergic neurons in lumbosacral spinal cord (de Groat, Booth, Milne, & Roppolo, 1982; de Groat, Vizzard, Araki, & Roppolo, 1996).These neurons can be identified by location and morphology, when choline acetyltransferase (ChAT) is used as an immunohistochemical marker of all spinal cholinergic neurons. Previous studies have found these neurons rarely show activity‐dependent transcription of c‐Fos, and in anesthetized rats c‐Fos+/ChAT+ neurons are not detected in the SPN under conditions where these neurons are known to be electrophysiologically active (Vizzard, 2000b). When this analysis was replicated in awake rats (Figure 5g–i) we found that c‐Fos is also not induced in sacral preganglionic neurons in the SPN following cystometry in awake female or male rats (both *n* = 6). In both sexes, c‐Fos^+^/ChAT^−^ neurons were most commonly situated in a region dorsal and adjacent to the main concentration of c‐Fos^−^/ChAT^+^ preganglionic neurons. This corresponded to the location of TH^+^ neurons.

### 3.5 c‐Fos co‐expression in Pax2+ inhibitory spinal cord neurons

**FIGURE 6:**
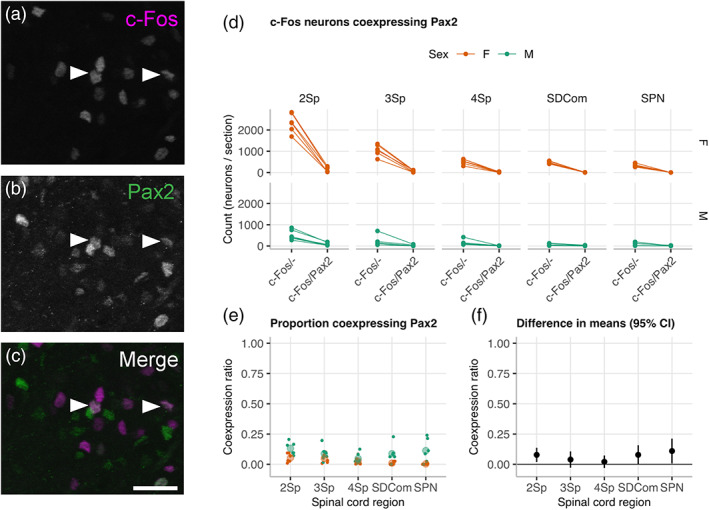
c‐Fos activation in inhibitory Pax2^+^ lumbosacral spinal cord neurons in male and female rats. (a–c) Images showing c‐Fos^+^ and Pax2^+^ immunolabeled nuclei (arrows) in neurons distributed in lamina II (2Sp) of a transverse section of L6 spinal cord in a male rat (scale bar = 50 μm). Plots comparing the L5–S2 rostrocaudal distribution of (d) c‐Fos^+^ and c‐Fos^+^/Pax2^+^ ROI neuron counts, and (e) ratios across 2Sp, lamina III (3Sp), lamina IV (4Sp) of the dorsal horn; sacral dorsal commissural nucleus (SDCom), and SPN after cystometry in female (*n* = 6) and male (*n* = 6) rats. Differences and 95% CI of group means in female and male groups (f) were not statistically significant. *P >* .05; Walsh Exact Test, Hommel correction for multiplicity [Color figure can be viewed at wileyonlinelibrary.com]

Pax2 is a marker for GABA inhibitory neurons in the dorsal horn of rat spinal cord (Todd, 2017). To determine if there was a sex difference in local inhibition of the dorsal horn following cystometry‐induced micturition, we used another series of sections from male rats in the same experiment for labelling with c‐Fos and Pax2 (Figure 6a–c). This showed the mean proportion of neurons classified as inhibitory across all regions was relatively low, ranging from 2 to 13% in males and 0 to 5% in females. No statistically significant difference in effect size was detected between sexes.

## 4 DISCUSSION

This study has used IEG activity mapping to analyze activation of the LUT‐related circuitry in lumbosacral spinal cord following cystometry‐induced micturition in awake rats of both sexes. By contrast, previous reports are mostly restricted to anesthetized female rats, and study effects of noxious LUT stimulation or models of bladder pathology such as cyclophosphamide‐induced cystitis. We therefore compared awake female and male rats to remove the effect of anesthesia. We also increased the spatial resolution of mapping and improve the sensitivity of the assay for analyzing effects of non‐noxious physiological activity in the LUT. We focused on the lumbosacral spinal cord as this contains the primary sensory and motor spinal cord projections that coordinate LUT reflex activities such as micturition and scent marking with complex behavioral output (Fowler et al., 2008; Holstege & Collewijn, 2009; Hou, Hyun, et al., 2016; Keller et al., 2018; Yao et al., 2018).

The experiments compared IEG activity mapping after cystometry‐induced micturition to a surgically naive control group. This experimental design has a technical limitation as it cannot detect baseline activation caused by the chronically implanted cannula when there is no cystometry. However, previous comparisons of surgically naive and sham operated groups (used as controls in acute cystometry studies performed under urethane anesthesia on female rats) did not detect any differential effects on c‐Fos^+^ neurons in lumbosacral spinal cord (Vizzard, 2000a, 2000b). This suggests the effect of implantation surgery is transient and consistent with the time course of activity‐dependent induction of c‐Fos and EGR‐1, which normally peaks around 2 hr and has a duration of around 6 hr (Yap & Greenberg, 2018). This time course does not appear to change significantly when rats recover from anesthesia after laparotomy and abdominal or other surgeries (Bojovic, Panja, Bittins, Bramham, & Tjolsen, 2015; Zittel, De Giorgio, Brecha, Sternini, & Raybould, 1993). A review of studies examining the effects of postimplantation time on cystometry and histological parameters (Andersson et al., 2011) shows large changes are typically measured on Days 1–3 but are substantially recovered by Day 7. Guided by these findings, we performed all testing on postsurgical Day 10. Our results, obtained by comparing surgically naive and cystometry groups of awake male and female rats, generally confirmed studies of anesthetized female rats comparing multiple control groups to cystometry‐induced micturition (Vizzard, 2000a, 2000b). We also found the effect of cystometry on c‐Fos neurons was larger in the lumbosacral spinal cord segments L6–S1 than thoracolumbar segments L1, L2, and L5; and the order of regional activation was SPN > SDCom > medial dorsal horn > lateral dorsal horn. To extend these findings, the present study increased spatial resolution in the rostrocaudal direction by using continuous ordered series of sections through segments L5–S2, and by using 11 spinal cord atlas regions for counting within each segment. As predicted by IEG mapping studies in anesthetized rats, our analysis of awake female, and male rats confirmed the SPN is activated following cystometry‐induced micturition. However, in contrast to these earlier reports, we found the SPN was less strongly activated than lamina II and III (2Sp and 3Sp) in dorsal horn. We also identified sex differences, as the dorsal commissural nucleus (SDCom) was only activated in female rats. Our results also suggested that cystometry‐induced micturition facilitated activation of 2Sp and 3Sp in female rats, but reduced activation of these dorsal horn regions in male rats.

Our results demonstrated cystometry‐induced micturition activation of the SPN in both sexes, but our results suggested that cystometry‐induced micturition had a greater effect in females. The SPN contains all sacral preganglionic neurons that project in the parasympathetic autonomic motor pathways to the LUT and other pelvic viscera (Anderson et al., 2009; Holstege & Collewijn, 2009). In rat, the primary pathway to the LUT is formed by SPN preganglionic neurons in spinal cord segments L6–S1 that innervate LUT‐projecting postganglionic neurons in the bilateral major pelvic ganglia. SPN preganglionic neurons provide the primary motor command that coordinates the activity of bladder smooth muscle (de Groat et al., 2015; Holstege & Collewijn, 2009). Their activity increases during the later phase of bladder filling, but these neurons are most active when they are contracting the bladder during voiding. Despite this known pattern of activity, preganglionic (ChAT^+^) neurons did not show induction of c‐Fos. However, this was consistent with reports that c‐Fos is induced in preganglionic neurons by cystometry following cyclophosphamide‐induced cystometry but is absent after cystometry in control groups (Vizzard, 2000a).

A major class of SPN neurons activated by cystometry‐induced micturition were identified as TH^+^ catecholamine neurons. This confirmed an earlier study (Hou, Carson, et al., 2016), which also found TH^+^ neurons in SPN were labeled with c‐Fos after cystometry. However, this was performed in anesthetized female rats and did not provide any spatial or quantitative analysis. Two lines of evidence suggest TH^+^ neurons in SPN function in regulating LUT activity (Hou, Carson, et al., 2016). First, TH^+^ neurons in SPN can be labeled by injecting transsynaptic tracer (pseudorabies virus) into the bladder wall. Second, cystometric activity in anesthetized rats is altered when TH^+^ neurons in SPN are lesioned with a chemical neurotoxin (6‐hydroxydopamine), consistent with evidence suggesting dopamine could be the primary neurotransmitter used by TH^+^ neurons in SPN (Hou, Carson, et al., 2016). The circuit functions of the remaining unidentified classes of ChAT^−^/TH^−^ SPN neurons activated by cystometry is mostly unknown, but there is recent evidence suggesting that some may coordinate LUT functions with somatic activity (Thor & de Groat, 2010). This is consistent with activation of the SPN detected by c‐Fos mapping following locomotion in decerebrate cats (Merkulyeva et al., 2019). Coordination of LUT and hindlimb motor activities was demonstrated by correlations in EMGs recorded in bladder detrusor, external urinary sphincter, and hindlimb muscles. These data suggest noncholinergic circuitry in the SPN could function in synchronizing the activation of the LUT with other distinct lumbosacral motor pools recruited by specific motor behaviors such as micturition and scent marking, or repressing LUT activity in the context of digestive voiding or sexual activity (Hou, Hyun, et al., 2016; Keller et al., 2018; Yao et al., 2018).

Our results suggest cystometry preferentially activated LTMRs in awake rats, as we saw relatively little activation of lamina I, which is targeted by second order visceral nociceptor neurons that project in the spinothalamic tract and are activated by high‐threshold mechanical and noxious chemical stimulation of the LUT (Palecek, Paleckova, & Willis, 2003). IEG activity mapping studies consistently report activation of lamina I in response to most forms of sustained nociceptor afferent activation, which includes high‐threshold or noxious stimulation in LUT or stimulation in models of peripheral LUT hyperalgesia (Birder & de Groat, 1992b, 1993; Mitsui et al., 2001; Mitsui et al., 2003; Vizzard, 2000a, 2000b). Physiological studies have determined that cystometry mostly activates A‐ and C‐LTMRs projecting to the LUT (Janig & Koltzenburg, 1990; Sengupta & Gebhart, 1994; Shea et al., 2000). It is this input that encodes the state of bladder filling and is used to initiate reflex voiding during cystometry. We found in females, that cystometry caused graded activation in a zone extending from lamina II to lamina IV. Within this zone, the rostrocaudal distribution of c‐Fos neurons peaked in spinal cord segment L6. This correlates with the L6 dorsal root ganglia providing the highest proportion of afferent projection to the LUT (Applebaum et al., 1980; Nadelhaft & Vera, 1996; Nance et al., 1988; Vera & Nadelhaft, 1992, 2000). In lumbar spinal cord, laminae II–IV functions as a low‐threshold mechanosensory receptor zone that processes LTMR afferent input from the hindlimbs (Abraira et al., 2017; Abraira & Ginty, 2013). Molecular anatomical studies have recently advanced our understanding of the functional organization of this circuitry and how it processes somatosensory LTMR input (Olson, Dong, Fleming, & Luo, 2016).

In awake male rats, the rostrocaudal distribution of c‐Fos neurons in Lamina II showed a peak in L6 following cystometry but not in the control group, whereas laminae II–IV showed a net inhibition of activity in dorsal horn following cystometry. Visualization of the regional and segmental distributions of c‐Fos suggested a relatively high baseline activity maintained by unstimulated awake male rats in the control group contributed to this apparent sex difference. To explore this further, we used EGR‐1 mapping as an alternate IEG activity marker to compare cystometry and control groups in male rats. Activation following cystometry‐induced micturition was detected in SPN by increased EGR‐1^+^ neurons but there was no change in dorsal horn EGR‐1^+^ neurons corresponding to the reduction in activity‐induced c‐Fos^+^ neurons. This discrepancy was explained by further analysis that showed activity‐dependent transcription of these IEGs in male rats was in two distinct but overlapping neural circuits. We have previously reported that c‐Fos and EGR‐1 can reveal different patterns of activity in response to noxious stimulation in rat after spinal cord injury (Densmore et al., 2010), and other many other groups also report differences in relative spatial distribution and number of c‐Fos and EGR‐1 neurons in the spinal cord following noxious visceral or somatic sensory stimulation (Herdegen, Rudiger, Mayer, Bravo, & Zimmermann, 1994; Ko et al., 2005; Lanteri‐Minet et al., 1993; Rahman et al., 2002).

Our data in awake male rats suggested cystometry‐induced micturition could engage local spinal cord inhibitory mechanisms that suppress the relatively high baseline dorsal horn activity seen in the control group. To explore this further we performed a coexpression analysis using Pax2 as a marker of inhibitory dorsal horn neurons. However, this demonstrated that expression of c‐Fos was strongly biased to excitatory (Pax2^−^) neurons in spinal cord dorsal horn. Another mechanism that could explain our observations is the engagement of descending inhibitory controls. These have been best described in the context of pain modulation, where noxious somatosensory input activates descending noxious inhibitory controls (DNICs; Le Bars, Dickenson, & Besson, 1979a, 1979b). This inhibition is suggested to act as a surround filter for non‐noxious somatosensory input to focus the perception of the noxious stimulus (Bannister & Dickenson, 2017; Le Bars & Willer, 2008). However, in the visceral sensory system, descending inhibitory controls can be engaged by both non‐noxious and noxious stimulation (Cadden & Morrison, 1991; Zhuo, 1992). This is clinically relevant, as bladder filling in humans attenuates somatomotor H‐reflexes and perception of nociceptive stimuli (Carbone et al., 2002; Inghilleri et al., 2001; Serrao et al., 2014). Also relevant to the present study is evidence from humans that bladder‐induced descending inhibition can be stronger in males than females (Fragiotta et al., 2015).

In conclusion, this study has shown that IEG mapping in awake rats can be used to extend our understanding of the functional molecular anatomy and identify sex differences in the LUT‐related circuit in spinal cord.

## Data Availability

Data supporting the findings of this study will be assigned a DOI and published under an open access license on sparc.science (RRID: SCR_017041).
